# Hip denervation: an approach for relieving pain, restoring function, and reducing inflammation in juvenile idiopathic arthritis patients

**DOI:** 10.1007/s10067-025-07490-0

**Published:** 2025-06-19

**Authors:** Khalifa A., Elsaman A. M., Hamed M., Aly H., Maaty A.

**Affiliations:** 1 Department of Rheumatology and Rehabilitation, Sohag University Hospital, Sohag, Egypt; 2 Department of Radiology, Sohag University Hospital, Sohag, Egypt; 3 Department of Rheumatology and Rehabilitation, Al-Azhar University, Al-Mokhayam Al-Dayem Nasr City, Cairo, Egypt; 4 Physical Medicine, Rheumatology and Rehabilitation, Suez Canal University, Ismailia, Egypt

**Keywords:** Hip denervation, Juvenile idiopathic arthritis

## Abstract

**Background:**

Juvenile idiopathic arthritis (JIA) frequently involves the hip joint, leading to significant pain, functional impairment, and long-term joint damage. Conventional treatment strategies, including pharmacologic therapy and intra-articular injections, may not provide adequate control. Hip denervation (HD) has emerged as a potential interventional approach for pain management in various musculoskeletal conditions, but its role in JIA-associated hip arthritis remains unclear. This study aimed to evaluate the effect of HD in JIA patients with unilateral hip arthritis on pain, function, and inflammatory parameters.

**Methods:**

One hundred twenty JIA patients were diagnosed according to the ILAR criteria with unilateral hip arthritis. They were assigned randomly into three groups: group 1 received hip denervation, group 2 received subcutaneous saline, and group 3 received intra-articular triamcinolone. Visual analog scale (VAS), sonography of large joints in rheumatology (SOLAR) score, tenderness, and Harris Hip score (HHS) were assessed at 0-, 2-, and 16-week intervals. Tenderness was evaluated by a semi-quantitative score at the same intervals. Juvenile Arthritis Disease Activity Score (JADAS) was assessed at baseline.

**Results:**

Over the 16-week study period, HD delivered remarkable outcomes, with VAS dropping from 5.48 ± 2.04 at baseline to 0.83 ± 0.50 (*p* < 0.0001), tenderness scores decreasing from 1.80 ± 0.82 to 0.80 ± 0.41 (*p* < 0.0001), and SOLAR score significantly reduced from 1.38 ± 0.59 to 0.15 ± 0.06 (*p* < 0.0001). Functional recovery was equally impressive, as HHS soared from 59.60 ± 9.89 to 83.27 ± 6.42 (*p* < 0.0001), surpassing outcomes seen with intra-articular steroids and placebo. Favorable responses were strongly associated with shorter disease duration, higher baseline VAS and SOLAR scores, and the oligoarticular subtype, while RF positivity predicted diminished improvement.

**Conclusion:**

Hip denervation showed promising results in regaining functions, alleviating pain, tenderness, and inflammation of the hip joint in JIA patients.

**Table:** 

Key Points • This study is the first to evaluate HD in JIA, showing its potential to alleviate pain, improve function, and reduce inflammation. • HD demonstrated sustained benefits at 16 weeks, surpassing intra-articular steroids in controlling symptoms and modifying the inflammatory cascade. • It offers a minimally invasive, effective alternative for JIA-related hip arthritis, addressing the limitations of conventional therapies

## Introduction

Juvenile idiopathic arthritis (JIA) encompasses seven subtypes according to the International League Against Rheumatism (ILAR) classification. In clinical practice, it is frequently divided into systemic and non-systemic, and they have different courses. Among these subtypes, hip arthritis is more prevalent in the systemic, polyarticular, and enthesitis-related forms [1]. Its incidence ranges from 20 to 60% [2] [3]. Chronic hip arthritis leads to irreversible joint destruction with impairment of life quality and functional limitation [4]. Hip damage occurs frequently as a complication of arthritis or steroid use. Early disease onset, early radiological changes, high doses of steroids for a long time, and higher baseline activity are all known risk factors that can predict hip joint damage in JIA [1].

Reserving nerve supply plays a critical role in maintaining the inflammatory cascade in JIA. Interestingly, hemiplegia has been observed to exert a protective influence against the destructive impact of rheumatoid arthritis (RA). The affected limb in hemiplegia experiences restricted blood flow, which may contribute to the suppression of inflammation. However, the precise mechanisms underlying this phenomenon remain unclear, warranting further investigation into whether impaired vascularity, impaired nerve supply, or a combination of both factors contributes to this intriguing effect. By untying these intricate mechanisms, we can deepen our understanding of JIA and potentially uncover novel therapeutic avenues for managing this complex condition [5–7]. Even though hip denervation (HD) has been used for short-term improvement of pain and function in advanced osteoarthritis (OA), after the failure of hip replacement, or unfitness for replacement, the application of HD in cases of inflammatory arthritis did not receive equivalent attention [8].

Our research group has conducted a series of clinical trials that have yielded promising outcomes regarding the potential of nerve block in alleviating various forms of inflammatory arthritis. These trials have brought forth favorable results. These exciting findings pave the way for further exploration and potential breakthroughs in the field of therapeutic approaches for inflammatory arthritis [9–11].

There is cumulative evidence that supports the anti-inflammatory role of local anesthetics. It is known that local anesthetics can suppress different inflammatory leukocyte functions including phagocytosis, adhesion, and migration [21]. Likewise, there is inhibition of different inflammatory leukotrienes and neurotransmitter release [10].

The scope of HD encompasses a diverse range of methods and employs various techniques. Traditionally, nerve block procedures have primarily served short-term purposes in pain management. However, interestingly, our previous research has revealed a contrasting scenario in the context of inflammatory arthritis. Specifically, nerve blocks have demonstrated superior and longer-lasting effects when employed in the management of inflammatory arthritis [12].

So far, the effect of sensory nerve block of active hip joints in JIA has not been assessed carefully. The present study is the first to explore this effect in the hip joints of JIA patients. We highlighted the impact of denervation on pain and function of inflamed hip joints compared to intra-articular steroids and placebo.

## Methods

### Patients

This study was approved by the ethical committee of the Faculty of Medicine Al-Azhar University (0000021). We enrolled JIA patients diagnosed after ILAR criteria with no condition for disease duration [13], with active unilateral hip arthritis aged ≥ 8 years at inclusion time. Hip arthritis was considered based on clinical examination or imaging. Written consent was signed by the study participants or their watchers to be included in the study and to publish the materials from the collected data. Participants with severe hip destruction and ankylosed hip, those receiving anticoagulant therapy, those with injection site infection, uncooperative participants, those with prior injection in the same joint in the last 6 months, or those who had an allergy to lidocaine were excluded from the study. All the participants were using disease-modifying anti-rheumatic drugs (DMARDs) and non-steroidal anti-inflammatory drugs (NSAIDs). A smaller percentage used biological treatment. All the systemic medications were not changed during the study. Further, all participants were informed thoroughly about the steps, objectives, and possible complications of the trial. The study was registered on clinicaltrials.gov number NCT04775225. All patient information was kept confidential. The study was consistent with the principles of the Declaration of Helsinki [14, 15].

### Randomization and blinding

Randomization was done using the 1:1:1 allocation ratio at the end of the baseline visit. The operators who conducted the interventions were unaware of the clinical data. The participants were blinded to the nature of the injected substance. The first author ensured clinical assessment and the initial ultrasound (US) evaluation, as well as the processes of randomization and blinding.

### Study design

**Fig. 1:**
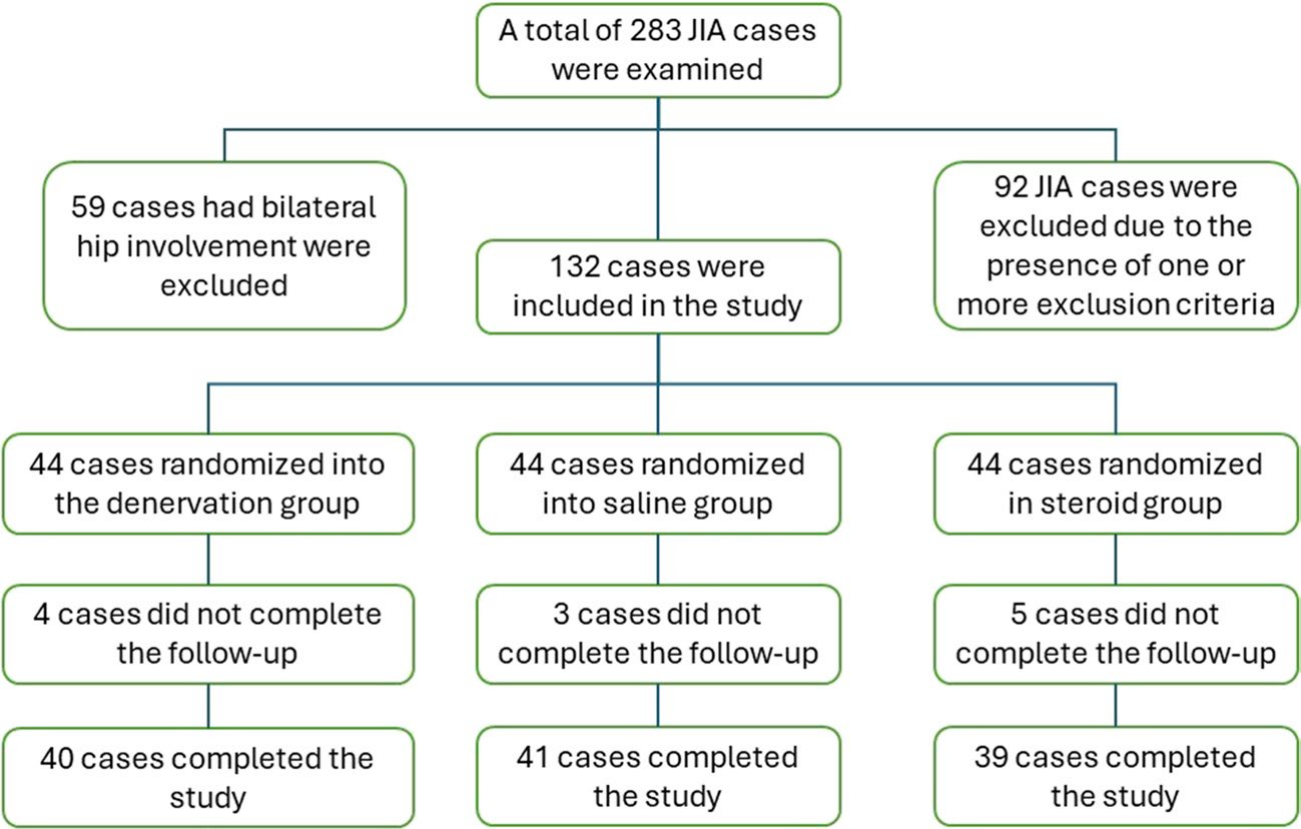
Flowchart of the study groups

Our study recruited participants exclusively from the rheumatology outpatient clinic, ensuring a homogeneous sample of JIA patients and unilateral hip involvement (Fig. 1). Comprehensive assessments were conducted at three distinct time points: baseline, 2 weeks, and 16 weeks. These evaluations encompassed a set of outcome measures including clinical examination, visual analog scale (VAS), Harris Hip score (HHS), semi-quantitative tenderness scoring, and sonography of large joints in rheumatology (SOLAR) score. Juvenile Arthritis Disease Activity Score (JADAS)-erythrocyte sedimentation rate (ESR) was performed at baseline only. To minimize potential statistical errors, participants with bilateral hip arthritis were excluded from the study. Additionally, we focused on children aged 8 years and older, considering the challenges associated with intervention in younger participants who may struggle to provide suitable responses to the questionnaires. HD, saline injection, and intra-articular steroid injection were all performed by two experienced sonographers.

### Hip denervation (HD)

We targeted the blockage of the articular branch of the femoral nerve and the articular branch of the obturator nerve. For the articular branch of the femoral nerve, participants were placed supine and the anterior inferior iliac spine and iliopubic eminence were marked. A 5–12 MHz linear probe (Toshiba APLIO 400 US system) was first positioned over the anterior superior iliac spine. Then, it was directed caudally to detect the anterior inferior iliac spine and the iliopubic eminence. Here, we can distinguish the iliopsoas muscle and tendon. A sterile technique was used for injection, and lidocaine spray was used at the injection site. A 25-gauge needle was used and inserted in-plane from lateral to medial deep to the iliopsoas tendon till the needle tip touched the pubic ramus. Aspiration was initially conducted to prevent intravascular injection, followed by injectate administration. While US provides real-time visualization of anatomical structures and needle placement, it does not definitively confirm whether the needle tip has inadvertently entered a small blood vessel. Fine capillaries or venules may not always be visible, particularly if Doppler settings are not optimized or if the vessel is temporarily collapsed. Aspiration serves as a critical precautionary step to verify accurate placement, thereby minimizing the risk of intravascular injection and potential systemic complications. As regards the articular branch of the obturator nerve, the probe was placed obliquely to reveal the femur head, neck, and superomedial acetabulum. The probe was then moved medial and inferior without changing direction till the femur head disappeared and the inferomedial acetabulum evolved. Another 25-gauge was inserted in-plane from laterally to medially till the needle touched the bone, and the same measures were like the first injection [16]. The probe was covered with sterile plastic during the whole procedure. Each nerve was injected with 2 mL of lidocaine hydrochloride 2% (Xylocaine, AstraZeneca). The vital signs were measured twice, before the procedure and half an hour after.

### Saline injection

Three milliliters of saline was injected in a single-point subcutaneous.

### Intra-articular steroid injection

The patient is positioned supine. The hip was in the neutral position. The knee was slightly flexed by a pillow underneath. A 5–12 MHz linear probe (Toshiba APLIO 400 US system) was used. The probe was put obliquely till the femur neck and head were identified. Then, the anterior joint recess was revealed at the junction between the head and neck. The sterile technique was used for injection, and lidocaine spray was used to minimize injection site pain. A 25-gauge needle was introduced from lateral to medial. A sterile 3.5-inch spinal needle was inserted using either an in-plane or out-of-plane technique, based on the specific preference and comfort of the individual [17]. We injected 4 mL of triamcinolone acetonide (TA) 80 mg (Kenacort, Bristol Myers Squibb). Doppler was used to warrant intra-articular injection [18]. Vital signs and blood sugar were measured before and after the procedure.

### Outcome measures

#### Visual analog scale (VAS) for pain

The VAS for the affected hip was also performed at 3 intervals. The VAS was mounted from 0 to 10. Grade 0 = no pain and 10 indicates the worst possible pain [19–21]. 

#### Harris hip score (HHS)

The HHS was used for hip function assessment at 0, 2, and 16-week intervals. This score was initially designed to assess the function after hip replacement. A score of 90–100: means excellent, 80–90: means good, 70–79 means fair, and 60–69: is poor function. It includes 4 subsets, pain (no pain was given 44 points), function (best score 33), function activities (best score 14), and physical exam (best score 9) [22].

#### Semiquantitative score for tenderness

The tenderness was assessed using a semi-quantitative score graded from 0 to 3; a score of 0 means no tenderness and 3 means maximum tenderness [23].

#### Juvenile arthritis disease activity score (JADAS)

The JADAS includes 4 domains: physician global assessment of disease activity (0 = no activity and 10 = maximum activity), parent/patient global assessment of well-being (0 = very well and 10 = very poor), number of active joints, and an inflammatory marker ESR. (ESR) (2). We used JADAS27. Value < 1 means inactive disease for all JIA categories. Minimal disease activity was 1 and 2 for oligoarticular JIA and 1 and 3.8 for polyarticular JIA. Children with systemic arthritis, rheumatoid factor (RF)– positive polyarthritis, RF-negative polyarthritis, or extended oligoarthritis were included in the polyarthritis group. The oligoarthritis group included patients with persistent oligoarthritis. Cutoff values for JADAS for “parent’s acceptable symptom state” were 2 and 3.2 for oligoarticular JIA and 3.8 and 5.2 for polyarticular JIA. Values above the cutoffs for “parent’s acceptable symptom state” were considered “active disease” states (JADAS 3.2 and JADAS 5.2 for oligoarticular and polyarticular JIA, respectively) [24].

#### SOLAR score

The SOLAR score was used to assess inflammation of the hip joint. The SOLAR score is a validated US-based assessment tool used to evaluate inflammatory and structural changes in large joints of patients with rheumatic diseases. This scoring system quantifies synovial inflammation, effusion, and structural damage, providing an objective measure of disease activity and response to treatment. All three groups underwent evaluation at 0, 2, and 16 weeks. A semi-quantitative 0–3 grey scale (GS) score was used. Grade 0 represents a normal joint with no detectable synovial hypertrophy or effusion, indicating the absence of inflammation. Grade 1 is characterized by mild synovial hypertrophy with a concave or flat joint capsule. In Grade 2, moderate synovial hypertrophy is present, accompanied by a convex joint capsule, which reflects a more pronounced effusion and early signs of capsular distension. Grade 3 denotes severe synovial hypertrophy, where the joint capsule adopts a barrel-shaped or markedly convex appearance due to substantial effusion and significant distension. The anterior longitudinal scan was considered. The same approach was used above for intra-articular hip injection [25, 26].

### Sample size calculation

We needed at least 37 patients in each group to reject the null hypothesis with a power of 0.80 (1-β error probability) and an α error probability of 0.05. We used G*Power software, version 3.1.9.7, for sample size calculation.

### Statistical analysis

The normality of the data was assessed using the Shapiro–Wilk test. Continuous variables were displayed as mean ± standard deviation (SD) or as median and range, depending on the normality of the data. Categorical variables were presented as frequencies and percentages. Comparisons between the three groups were performed using one-way analysis of variance (ANOVA) for normally distributed variables, followed by post hoc Tukey tests to identify pairwise differences. The Kruskal–Wallis test was utilized, succeeded by Dunn’s tests for conducting multiple comparisons among non-parametric variables. Repeated measures ANOVA was employed to assess temporal changes within each group at the baseline, 2-week, and 16-week time points. Pearson’s chi-squared or Fisher’s exact test was used to analyze categorical data. Statistical significance was set at *p* < 0.05, and all analyses were performed using SPSS software version 25.0 (IBM Corp., Armonk, NY). This rigorous approach ensured robust comparisons and reliable interpretation of the study outcomes.

## Results

The age of the study population ranged from 8.2 to 15.6 years without significant differences between groups. All patients had established disease with more than 1-year duration. Mean JADAS (± SD) were 3.97 ± 1.83, 3.81 ± 1.59, and 4.04 ± 1.50 in HD, control, and TA groups, respectively (*p* > 0.05). The females comprised around 60% of all groups. The positivity of RF ranged from 25 to 50% in the studied patients. The frequency of right hip affection was 57.5% in the HD group, 78% in the control group, and 61.5% in the TA group. Approximately 34% of patients were administered steroids in their medications. Subtypes of JIA in our study include oligoarticular (43.3%), polyarticular (23.3%), enthesitis-related (22.5%), and systemic type (10.8%). In terms of patients’ characteristics, no statistically significant differences were found between groups (*p* > 0.05), as shown in Table 1.

**Table 1:** Patients’ characteristics in the study groups

Variables	HD (*n* = 40)	Control (*n* = 41)	TA (*n* = 39)	*p*-value
Age (yr), median (range)	12.6 (8.5–15.6)	12.9 (8.2–15.4)	12.4 (9.1–15.4)	0.961
Duration (yr), median (range)	5.40 (2.5–7.1)	4.57 (1.5–6.9)	5.20 (2.3–7.1)	0.054
JADAS, mean ± SD	3.97 ± 1.83	3.81 ± 1.59	4.04 ± 1.50	0.814
Female, *n* (%)	24 (60.0%)	26 (63.4%)	23 (59.0%)	0.913
RF positive, *n* (%)	21 (52.5%)	13 (31.7%)	10 (25.6%)	0.125
Right-sided hip, *n* (%)	23 (57.5%)	32 (78.0%)	24 (61.5%)	0.117
Steroids, ***n*** (%)	17 (42.5%)	8 (19.5%)	16 (41.0%)	0.051
JIA subtypes				0.304
Oligoarticular, *n* (%)	18 (45.0%)	20 (48.7%)	14 (35.9%)	
Polyarticular, *n* (%)	7 (17.5%)	11 (26.8%)	10 (25.6%)	
Enthesitis-related, *n* (%)	12 (30.0%)	4 (9.8%)	11 (28.2%)	
Systemic, *n* (%)	3 (7.5%)	6 (14.6%)	4 (10.3%)	

*JADAS* Juvenile Arthritis Disease Activity Score (JADAS), *RF* rheumatoid factor

**Fig. 2:**
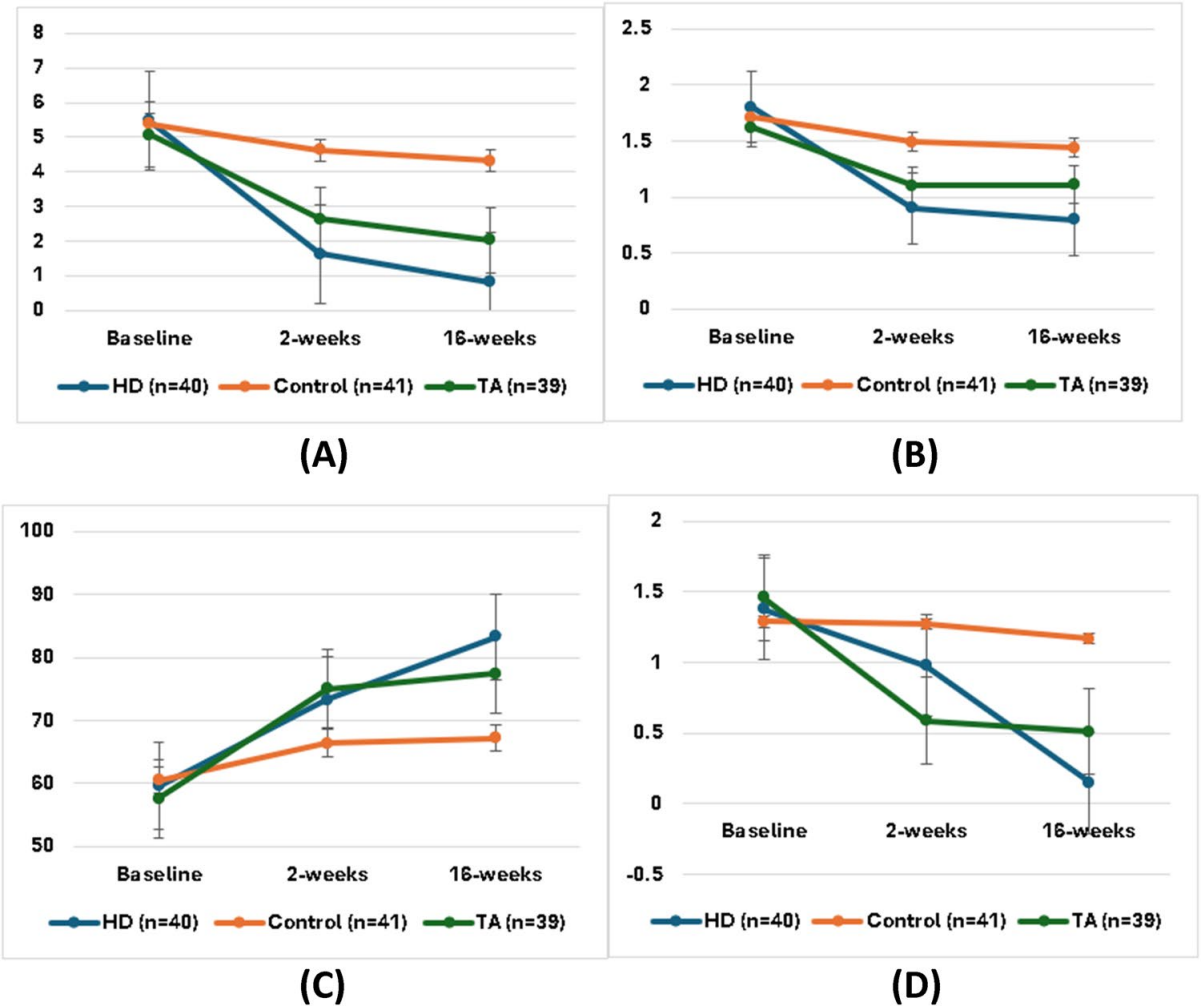
Outcome measures during follow-up in the study groups: **A** VAS, **B** tenderness score, **C** HHS, and **D** SOLAR score

Table 2 and Fig. 2 illustrate the outcome measures recorded during the follow-up periods for the participants involved in the study. The analysis indicated that both VAS and tenderness scores experienced a significant decrease 2 weeks post-intervention in the HD and TA groups compared to the placebo group, with the HD group demonstrating a greater level of significance than the TA group (*p* < 0.05). In contrast, the SOLAR score at the 2-week time point exhibited a notable reduction in both the HD and TA groups, with the TA group showing a higher level of significance relative to the HD and control groups (*p* < 0.05). Furthermore, the HHS functional assessment revealed a significant improvement in the denervation and steroid groups 2 weeks after the intervention, with the TA group showing a more pronounced significance when compared to the control group (*p* < 0.05). After 16 weeks, the VAS, tenderness, and SOLAR scores were considerably lower in the HD group compared to the TA and control groups (*p* < 0.05). The HHS at 16-week intervals was higher in the HD and TA groups than in the control group (*p* < 0.05).

**Table 2:** Outcome measures in the study groups

Variables	HD (*n* = 40)	Control (*n* = 41)	TA (*n* = 39)	*p*-value
VAS				
Baseline	5.48 ± 2.04	5.39 ± 1.80	5.08 ± 1.78	0.733
2 weeks	1.63 ± 0.59^2,3^	4.63 ± 1.73	2.64 ± 1.42^2^	< 0.0001
16 weeks	0.83 ± 0.50^2,3^	4.32 ± 1.71	2.03 ± 1.37^2^	< 0.0001
* p*-value	< 0.0001	0.045	< 0.0001	
Tenderness				
Baseline	1.80 ± 0.82	1.71 ± 0.75	1.62 ± 0.67	0.661
2 weeks	0.90 ± 0.30^2^	1.49 ± 0.55	1.10 ± 0.31^2^	< 0.0001
16 weeks	0.80 ± 0.41^2,3^	1.44 ± 0.50	1.11 ± 0.29^2^	< 0.0001
* p*-value	< 0.0001	0.272	< 0.0001	
HHS				
Baseline	59.60 ± 9.89	60.54 ± 10.98	57.61 ± 10.55	0.403
2 weeks	73.24 ± 6.61	66.42 ± 14.11	75.07 ± 11.98^2^	0.003
16 weeks	83.27 ± 6.42^2^	67.22 ± 14.92	77.45 ± 13.20^2^	< 0.0001
* p*-value	< 0.0001	0.020	< 0.0001	
SOLAR				
Baseline	1.38 ± 0.59	1.29 ± 0.51	1.46 ± 0.68	0.579
2 weeks	0.98 ± 0.48	1.27 ± 0.55	0.59 ± 0.03^1,2^	0.001
16 weeks	0.15 ± 0.06^2,3^	1.17 ± 0.86	0.51 ± 0.06^2^	< 0.0001
* p*-value	< 0.0001	0.464	< 0.0001	

^1^Significant *p*-value < 0.05 versus group 1 (HD group), ^2^significant *p*-value < 0.05 versus group 2 (control group), ^3^significant *p*-value < 0.05 versus group 3 (TA group), *HD* hip denervation, *TA* triamcinolone acetonide, *VAS* visual analog scale, *HHS* Harris Hip score, *SOLAR* sonography of large joints in rheumatology

As shown in Table 3, predictors of good response were shorter disease duration, greater pain, tenderness, SOLAR scores, and oligoarticular JIA, while poor predictors include positivity of RF.

**Table 3:** Regression analysis assessing predictors of response of JIA to injections

Variables	*B*	Std. error	Beta	*t*	*p*-value
(Constant)	− 0.119	0.672		− 0.176	0.860
Age	0.041	0.035	0.107	1.169	0.245
Sex	0.107	0.132	0.071	0.813	0.418
Duration	− 0.130	0.053	− 0.226	− 2.434	0.017
RF positivity	− 0.788	0.376	− 0.167	− 2.093	0.039
Steroids administration	− 0.125	0.137	− 0.080	− 0.915	0.362
JADAS	− 0.078	0.114	− 0.056	− 0.682	0.497
Baseline VAS	− 0.733	0.092	− 0.599	− 7.991	< 0.0001
Baseline tenderness score	− 1.764	0.229	− 0.578	− 7.692	< 0.0001
Baseline HHS	0.002	0.006	0.030	0.343	0.733
Baseline SOLAR score	− 0.407	0.108	− 0.325	− 3.766	< 0.0001
Oligoarticular JIA	0.303	0.116	0.263	2.616	0.010
Polyarticular/systemic JIA	0.183	0.119	0.141	1.546	0.125
Enthesitis-related JIA	0.203	0.439	0.046	0.463	0.644

*RF* rheumatoid factor, *JADAS* Juvenile Arthritis Disease Activity Score (JADAS), *VAS* visual analog scale, *HHS* Harris Hip score, *SOLAR* sonography of large joints in rheumatology, *JIA* juvenile idiopathic arthritis

As indicated in Table 3, factors associated with a favorable response included a shorter duration of the disease, increased levels of pain and tenderness, higher SOLAR score, and oligoarticular JIA. Conversely, a positive RF was identified as a negative predictor.

Insignificant minor side effects were observed across all three groups. Within the first (HD) group, two patients (5.0%) reported temporary numbness that resolved spontaneously without medical intervention. Conversely, in the control group, one individual (2.4%) encountered a mild vasovagal episode. This vagal response was managed effectively through rest, leg elevation, gradual consumption of isotonic fluids, and counterpressure methods, including handgrip and leg crossing. In the TA group, one case (2.6%) developed hematoma at the injection site and another case (2.6%) experienced localized pain in the joint where the injection was administered. These side effects were effectively managed through local rest, the application of ice packs, and the use of mild analgesics.

## Discussion

Hip destruction occurs relatively early in JIA. Existing therapies showed disappointing results in preventing hip joint damage. Steroid use in JIA, either intra-articular or systemic, was associated with a relative risk of avascular necrosis. In many patients, escalating systemic treatment failed to control monoarthritis. Although nerve block demonstrated favorable outcomes in managing pain, inflammation, and functionality in cases of JIA with persistent knee arthritis, HD was not evaluated in the same group of patients. This study constitutes the first investigation of this effect compared to placebo and intra-articular steroid injection [27–29].

**Fig. 3:**
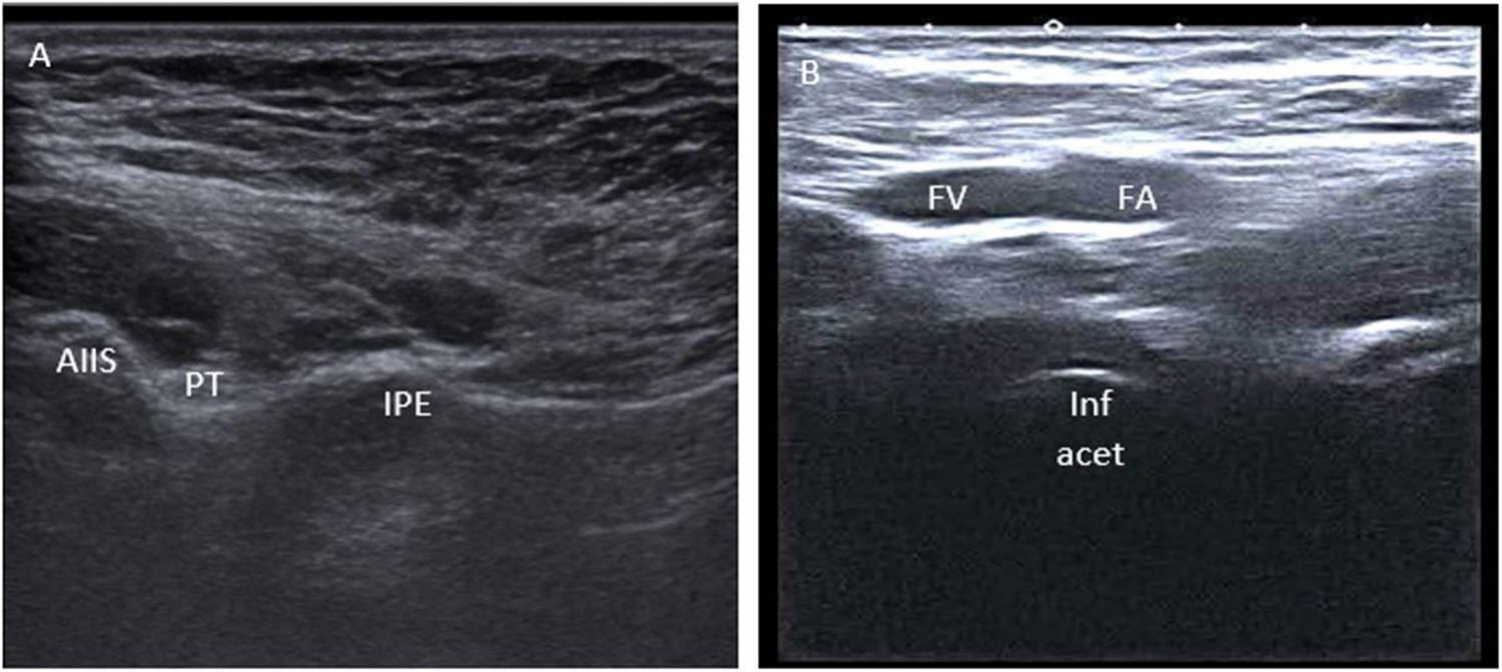
The sonoanatomical landmarks of the hip articular branches, highlighting the femoral bracn at the level of the iliopectineal eminence (**A**) and the accessory obturator nerve at the level of the inferomedial acetabulum (**B**). Key anatomical structures labeled in the figure include the anterior inferior iliac spine (AIIS), psoas tendon (PT), iliopectineal eminence (IPE), femoral vein (FV), femoral artery (FA), and inferomedial acetabulum (Inf acetab)

In the present study, HD significantly alleviated pain and tenderness after 2 weeks compared to the other two groups. Steroid injections demonstrated notable enhancements in the function, tenderness, and SOLAR scores at the 2-week mark. After 16 weeks, HD exhibited markedly better results across all measured parameters. Particularly, the HD group expressed a prolonged period of decline, lasting until 16 weeks, with only a minimal rebound in the outcome measures to the pre-intervention values (Fig. 3).

Shorter disease duration and higher baseline VAS and tenderness scores were associated with better outcomes, while positive RF predicted a worse response. Patient age, sex, and baseline JADAS did not significantly influence improvement in the studied groups.

The impact of HD on the inflammatory markers assessed by the SOLAR score introduces a new dimension to its therapeutic potential. Our findings indicate a notable decrease in synovial inflammation, as evidenced by US assessments, whereas earlier research has mainly concentrated on pain and functional outcomes. These results specify that HD reduces symptoms and modifies the underlying disease process. The anti-inflammatory properties observed merit additional research, especially compared to systemic therapies, for a deeper understanding of its mechanisms and long-term effects on disease progression.

While pain in inflamed joints may serve a protective role by limiting mechanical stress and preserving cartilage integrity, excessive immobilization can lead to muscle atrophy [30]. Chronic inflammation, rather than mechanical load alone, remains the primary driver of joint destruction in JIA [31]. Concerns have been raised about NSAIDs, particularly diclofenac, potentially accelerating cartilage degradation through prostaglandin inhibition, though evidence remains inconclusive [32]. HD offers a targeted approach that alleviates pain while reducing inflammation, as reflected in sustained improvements in SOLAR scores, without disrupting systemic prostaglandins. By addressing both pain and inflammation, HD may provide a safer, function-preserving alternative to prolonged NSAID use in JIA.

In a previous study by Radwan et al., they found that after 2 weeks, genicular nerve block (GNB) improved pain and tenderness better than intra-articular steroids when injected in persistent knee arthritis of JIA patients. On the same point, steroids regained better function and enhanced SOLAR scores. The GNB group showed better results in all the outcome measures after 12 weeks [28]. Those results are agreeable with ours. In the present study, denervation showed better but comparable results with steroid injection. The exact mechanism by which joint denervation works is not fully understood. Nerve block improves pain rapidly by directly influencing the sensory supply of the joint. The improvement of function and inflammation lagged more. It is known that anesthesia can inhibit leukocyte adhesion, phagocytosis, degranulation, cytokine release, and migration [33]. Anesthesia can suppress adrenergic proinflammatory neurotransmitters [34, 35]. Those neurotransmitters control macrophages’ behavior through receptors on their surface [35]. Botulinum toxin A improves persistent synovitis and swelling of the ankle joint after the failure of other modalities. This effect was noticed after 2 weeks of injection [36]. This indicates that anesthesia needs time to control inflammation through several mechanisms. Further, this could justify the delayed impact of HD on the control of SOLAR and HHS.

Radiofrequency denervation controls pain and improves function for about 6 months in hip OA [37, 38]. This technique for denervation was used in adults with a degenerated joint for long-term control of pain only and was not approved in pediatrics. After the promising results of GNB in JIA, we assumed that the same technique could yield good results with no need for radiofrequency [12].

Lidocaine has a relatively short half-life. It is nearly 2 h [39]. Lidocaine can reduce pain, improve function, and decrease inflammation for 16 weeks, although the underlying reasons for this effect remain unclear. The present study assumes that HD was able to break the inflammatory cascade, and it required 16 weeks at least for retrieval [28].

The hip injection in JIA did not get the same consideration as the knee joint. Only steroid injection was tested in the hip joint. No clinical trials are available for methotrexate, anti-TNF, and Botox injections in the hip joint of JIA patients. In literature, there is great controversy about the cause of avascular necrosis in the femoral head of JIA patients. There is no consensus on whether it is related to the disease activity or intra-articular steroid injection [40, 41]. The steroid could control joints and function for at least 12 weeks. This improvement varied deeply depending on the disease pattern. The oligoarticular form showed the best response as in our study [42]. The hip joint showed the worst response to intra-articular steroids among injected lower limb joints. Destruction persisted in half of the injected joints [43].

It was noticed that steroid injection can improve pain and function in knee OA for only 2 weeks. This effect extends for 3 months when injected in the knee of adult RA patients and could reach up to 9 months when injected in the knee of JIA patients. This assumes that the steroid effect runs parallel to the degree of inflammation. A similar effect was noticed in the GNB of the knee in OA and RA [12].

The results of this study have significant implications for advancing the management of JIA-related hip arthritis, offering a minimally invasive yet effective alternative to conventional treatments. The ability of HD to achieve sustained improvements in pain, function, and inflammation, even outstanding intra-articular steroids at 16 weeks, highlights its potential to shift clinical practice toward targeted, joint-specific interventions. Moreover, by demonstrating HD’s capacity to break the inflammatory cascade, this study introduces a paradigm that extends beyond symptomatic relief to possibly influencing disease mechanisms. These findings contribute to the growing body of evidence supporting nerve block therapies and open new avenues for integrating HD into comprehensive JIA treatment frameworks, making it a compelling option for rheumatologists and interventional pain specialists.

### Limitations of the study

Gait analysis before and after injection could add great value to the present study. Unfortunately, our university did not have a specialized unit for gait analysis. Although triamcinolone hexacetonide has a longer-lasting effect than acetanoide, it is not available in Egypt.

## Conclusion

HD can control pain and tenderness, improve function, and mitigate inflammation in JIA. This effect could extend for 16 weeks with a significant difference in comparison to baseline and intra-articular steroids. HD is an excellent alternative for managing hip arthritis in JIA with minimal adverse effects.
